# Retraction: Inhibited corneal neovascularization in rabbits following corneal alkali burn by double-target interference for VEGF and HIF-1α

**DOI:** 10.1042/BSR-2018-0552_RET

**Published:** 2024-05-31

**Keywords:** Alkali burn, HIF-1α, Rabbit model, VEGF

This article is being retracted from *Bioscience Reports* at the request of the Editor-in-Chief and the Editorial Board following receipt of a notification from a reader, alerting the Editorial Board to concerns surrounding the validity of the loading controls used in the Western blots presented in Figure 6A and Figure 6B. The Editorial Office also have additional concerns regarding undeclared replacement of the images used in Figure 6A and Figure 6B during the manuscript revision process, and that these Western blots have the appearance of a composite image. The authors have been contacted with regards to the retraction and have not responded to the Journal's queries or the concerns raised. Given the concern surrounding the scientific validity of this paper, the Editorial Board stand by the decision to retract the article.

